# Defect Imide Double Antiperovskites *AE*_5_As*Pn*(NH)_2_ (*AE*=Ca, Sr; *Pn*=Sb, Bi) as Potential Solar Cell Absorber Materials

**DOI:** 10.1002/anie.202500768

**Published:** 2025-02-25

**Authors:** Thanh G. Chau, Dan Han, Florian Wolf, Stefan S. Rudel, Yuxuan Yao, Harald Oberhofer, Thomas Bein, Hubert Ebert, Wolfgang Schnick

**Affiliations:** ^1^ Department of Chemistry and Center for NanoScience (CeNS) University of Munich (LMU) Butenandtstr. 5–13 81377 Munich Germany; ^2^ School of Materials Science and Engineering Jilin University Changchun 130012 China; ^3^ Theoretical Chemistry and Catalysis Research Center Technical University Munich Lichtenbergstr. 4 85747 Garching Germany; ^4^ Theoretical Physics VII University of Bayreuth Universitätsstr. 30 95447 Bayreuth Germany

**Keywords:** ammonothermal synthesis, lead-free perovskite, photovoltaics, antiperovskite derivatives

## Abstract

An abundance of oxide, halide and chalcogenide perovskites have been explored, demonstrating outstanding properties, while the emerging nitride perovskites are extremely rare due to their challenging synthesis requirements. By inverting the ion type in the perovskite structure, the corresponding antiperovskite structure is obtained. Among them, ternary antiperovskite nitrides *X*_3_ *A*N (*X*=Ba, Sr, Ca, Mg; *A*=As, Sb) have recently been identified as exhibiting excellent optoelectronic properties. To explore the unrealized composition space of nitride perovskites, the ammonothermal method was applied, yielding three new layered quaternary imide‐based defect‐antiperovskites, namely *AE*_5_As*Pn*(NH)_2_ (*AE*=Ca, Sr; *Pn*=Sb, Bi). These new compounds feature distorted square‐pyramidal coordination around the imide‐group (Ca_5_NH). Layers with Ca^2+^ vacancies are found with an alternating As^3−^ and *Pn* ^3−^ (*Pn* ^3−^=Sb^3−^, Bi^3−^) coordination along the *A*‐site, forming a two‐dimensional (2D) structure. All three *AE*_5_As*Pn*(NH)_2_ compounds show suitable direct band gaps within the visible light spectrum. Density functional theory calculations reveal favorable band dispersion, as well as transport and optical properties, especially along the out‐of‐plane direction, demonstrating their 3D character of electronic transport. The narrow tunable direct band gaps and favorable charge carrier properties make *AE*_5_As*Pn*(NH)_2_ promising candidates for solar cell absorber materials.

## Graphical abstract

The ammonothermal method enabled the synthesis of antiperovskites with NH^2−^‐groups instead of N^3−^‐ ions, which introduces vacancies and replaces the octahedral unit by square‐pyramidal units. These imide‐based defect‐antiperovskites *AE*_5_As*Pn*(NH)_2_ (*AE*=Ca, Sr; *Pn*=Sb, Bi) showcase a new route to structurally engineer antiperovskites with cation‐deficiencies. Their direct band gaps have been confirmed and density functional theory calculations revealed good band dispersion, transport properties and optical properties suitable for solar cell absorber materials.

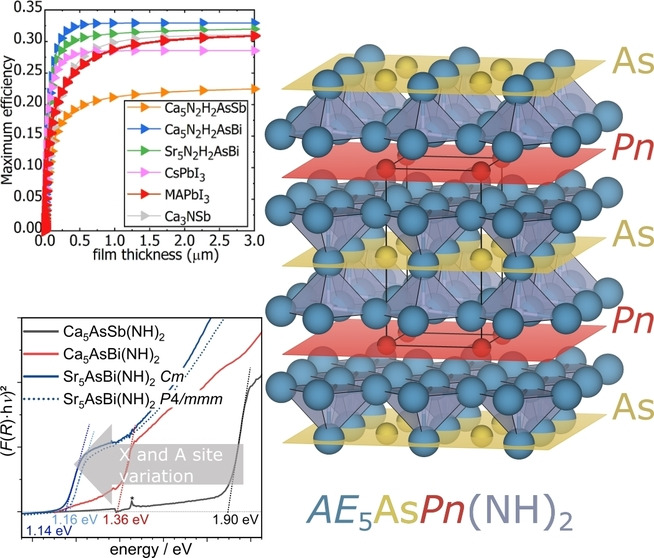

## Introduction

Halide perovskites have attracted significant interest due to their appealing optoelectronic properties and facile synthesis.[^1^, ^2^] However, concerns regarding lead‐related toxicity and stability have driven substantial efforts to find lead‐free alternatives.^[3]^ Chalcogenide perovskites have been proposed as one solution.[^4^, ^5^] In particular, Zr‐chalcogenide perovskite materials are found to be thermally stable against moisture and oxidation,^[6]^ which is a major advantage over the halide perovskites. Although first thin film samples of BaZrS_3_ have been prepared, further optimization of the thin film quality is still required.[^7^, ^8^] In contrast to the intense research on halide perovskites and chalcogenide perovskites, nitride perovskites remain less explored. Although nitride perovskites are interesting as they could easily integrate with a large number of nitride‐based semiconducting devices being used already, only few have been discovered so far due to the synthetic challenge of overcoming the stable N_2_ triple bond (+945 kJ/mol) and the low electron affinity of nitrogen (0.07 eV).[^9^, ^10^, ^11^] However, recently growing interest led to the successful synthesis of nitride perovskites such as LaReN_3_, CeMoN_3_, CeWN_3_ and LaWN_3_, with LaWN_3_ even been obtained as oxygen‐containing thin films.[^12^, ^13^, ^14^] Nevertheless, theoretical studies hint to inferior optoelectronic and defect properties of LaWN_3_ compared with its halide counterparts.^[9]^ Fully inorganic oxide and nitride antiperovskites, however, have recently gained research attention due to their promising physical properties, including thermoelectric performance,[^15^, ^16^, ^17^] optoelectronic potential,[^18^, ^19^, ^20^] and ionic conductivity,^[21]^ making them suitable for applications such as inorganic electrolytes for all‐solid‐state batteries^[22]^ or high‐temperature ceramics.[^23^, ^24^] The antiperovskite (X_3_AB) structure is related to the perovskite (ABX_3_) structure, but with the ion type inverted. Despite the theoretically large variety of possible antiperovskite compounds, only few studies on these compounds exist, especially compared to the conventional halide perovskites, due to the high air‐ and moisture sensitivity of the materials, which currently proves to be a challenge for thin film preparation. Mg_3_SbN is so far the only reported example of a thin film with antiperovskite structure, where the films have been encapsulated in AlN to prevent atmospheric oxidation and hydrolysis.^[20]^ Nevertheless, the fully inorganic nitride antiperovskites *AE*_3_ *Pn*N (*AE*=Mg, Ca, Sr; *Pn*=P, As, Sb, Bi) were found to exhibit direct band gaps, with highly dispersive valence bands (VB) and conduction bands (CB) and high absorption coefficients.[^18^, ^19^] Inspired by the double perovskites, anion ordering and anion mutation have been introduced into the ternary nitride and oxide antiperovskites, leading to the formation of quaternary antiperovskite nitrides with the formula *X*_6_ *AA*’*B*_2_ and *X*_6_ *A*_2_ *BB*’ (*X*=Mg, Ca, Sr, and Ba) where *A*‐ and *B*‐site anions are being group V elements, and *X*_6_NFSn_2_ (*X*=Ca, Sr, Ba).[^15^, ^18^] Among them, the Ca_6_AsSbN_2_ compound with *P*4/*mmm* symmetry shows a layered A‐site arrangement where density functional theory (DFT) calculations revealed optoelectronic properties on par with MAPbI_3_.^[18]^ Other observed A‐site arrangements are a rock salt or columnar ordering pattern, which are both rare cases.^[25]^ In addition to the theoretical prediction, however, most of the experimentally reported quaternary antiperovskites with a layered motif are based on the anti‐Ruddelsden‐Popper phases which were first described as antiperovskites by Gäbler et al.[^26^, ^27^, ^28^] There are also a limited number of compounds containing layered antiperovskite structure motifs, which can be derived from “classical” antiperovskites, like Sr_7_Sn_3_N_2_ ^[29]^ where [Sr_7_SnN_2_]^8+^ antiperovskite slabs are separated by [Sn_4_]^8−^ Zintl anions or Sr_11_Ge_4_N_6_ ^[30]^ which is similarly composed but separated by sheets of bent [Ge^II^N_2_]^−^ ions and layers of [Sr_4_Ge]^4−^. However, all of them are metallic, making them unsuitable for photovoltaic applications. Interestingly, a potential route for discovering new semiconducting layered structures would be to introduce vacancies on the *X*‐site, where a layered alternate stacking of two different ions has been induced on the *A*‐site, similarly to PrBaCo_2_O_5_.^[31]^ For the synthesis of such oxide perovskites, two methods have already been established to introduce vacancies. The first method introduces vacancies during the synthesis using elements suitable for doping or by changing the synthesis conditions. The other method uses a post‐treatment, such as acidic‐ and thermal treatments under a reducing environment. Both methods have been extensively investigated, as oxygen vacancies can play a crucial role for catalytic reactions like CO‐oxidation or oxygen evolution reaction (OER).[^32^, ^33^] Here, we introduce a new method to structurally engineer antiperovskite‐related compounds. Using the ammonothermal method, NH^2−^‐groups replace the N^3−^ ions on the *B*‐site, which introduces vacancies on the *X*‐site for compensation of charge and structural distortion. This route enables the synthesis of novel cation‐deficient antiperovskites.

In this work, an experimental and theoretical joint study of a series of three novel layered quaternary imide‐based defect‐antiperovskites, namely Ca_5_AsSb(NH)_2_,

Ca_5_AsBi(NH)_2_, and Sr_5_AsBi(NH)_2_, with direct band gaps is reported. The ammonothermal method has been employed for their experimental synthesis. An *A*‐site ordered structure was achieved by the NH^2−^‐group and the two different ion sizes of As^3−^ and *Pn* ^3−^ (*Pn*=Sb, Bi), which lead to vacancies on the *X*‐site as one *AE* ^2+^ is replaced by two H^+^, thereby preserving charge neutrality. The *AE*_5_As*Pn*(NH)_2_ compounds (*AE*=Ca, Sr; *Pn*=Sb, Bi) crystallize in the tetragonal space group *P*4/*mmm*. Moreover, when changing the synthesis conditions, monoclinic phases have been discovered for Ca_5_AsSb(NH)_2_ and Sr_5_AsBi(NH)_2_, crystallizing in space group *Cm*. Their crystal structures were solved and refined by single‐crystal X‐ray diffraction (scXRD) and powder X‐ray diffraction (pXRD) with an *AE*/*Pn* atomic ratio of 5/2, which could also be confirmed by energy‐dispersive X‐ray (EDX) spectroscopy measurements. Further investigations of the crystal structure of the three compounds were carried out by ^1^H‐magic angle spinning nuclear magnetic resonance (MAS‐NMR), IR‐ and Raman‐spectroscopy. DFT calculations of electronic structure, optoelectronic properties and charge transport properties were performed as well. The optical properties of the novel antiperovskites were studied by UV/Vis‐spectroscopy and by means of calculated absorption spectra, revealing narrow direct band gaps making the discovered compounds promising as solar cell absorber materials.

## Results and Discussion

### Synthesis and Crystal Structure

**Figure 1:**
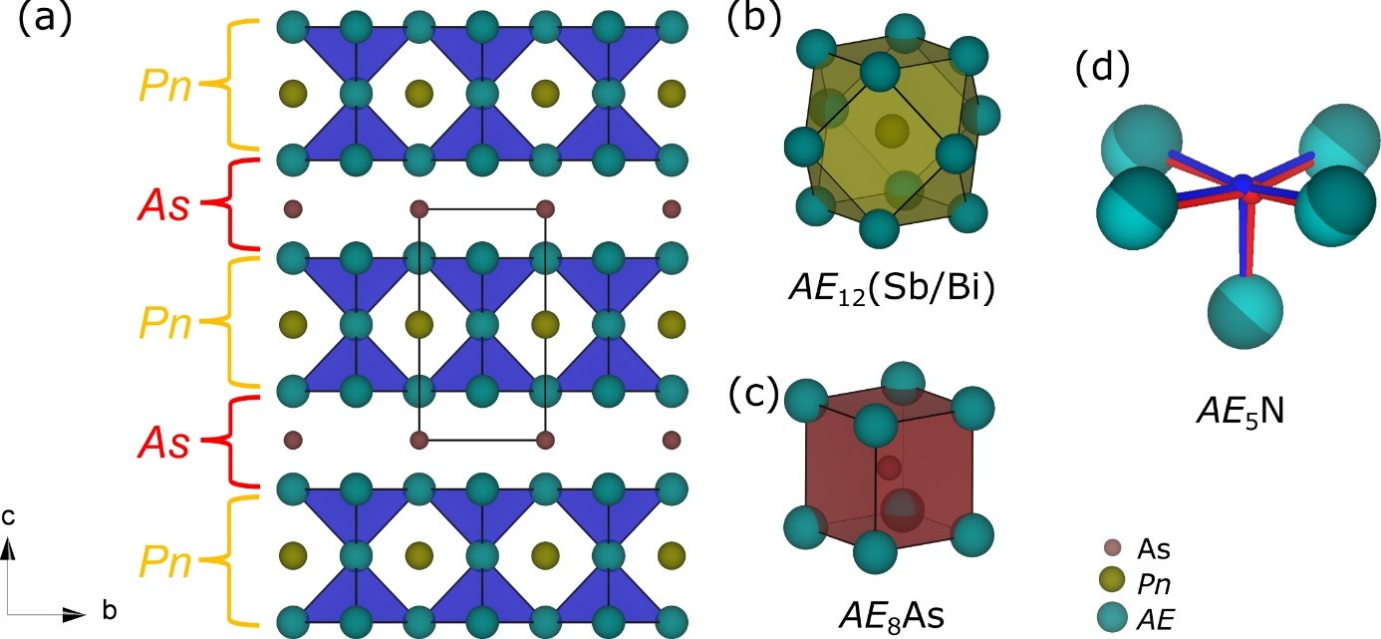
Crystal structure of *AE*_5_As*Pn*(NH)_2_. (a) Projection along [100], (b) icosahedral coordination polyhedron of Sb and Bi, (c) cube‐like coordination of As, (d) square‐pyramidal *AE*_5_NH unit with the distortion in *Cm* overlaid in red.

The compounds Ca_5_AsSb(NH)_2_, Ca_5_AsBi(NH)_2_ and Sr_5_AsBi(NH)_2_ were synthesized using the ammonothermal method in custom–made autoclaves from Haynes® 282® superalloy.^[34]^ KN_3_ was used as an ammonobasic mineralizer precursor for the single crystal growth of Ca_5_AsSb(NH)_2_ as it reacts to KNH_2_ with NH_3_ under the applied conditions which increases the solubility of the starting materials through formation of soluble species such as KCa(NH_2_)_3_.^[35]^ The bismuth‐containing compounds were synthesized without mineralizers as the addition of alkali sources would lead to the formation of byproducts *A*Bi_2_ (*A*=K, Rb, Cs).^[36]^ Reactions have been conducted with *Pn*/*AE* atomic ratios between 1 : 2.5 and 1 : 3 where the increased *AE* content lead to the formation of additional *AE*(NH_2_)_2_ in the colder zone of the reaction vessel, which could be separated from the bulk material. A heating temperature of 1020–1070 K was applied for 30–40 h with autogenous ammonia pressure between 100 and 140 MPa. The crystal structures of all three compounds *AE*_5_As*Pn*(NH)_2_ were solved and refined from single‐crystal X‐ray diffraction data (SI Tables S1–S7) (scXRD) in the tetragonal space group *P*4/*mmm* (no. 123). A second modification crystallizing in the monoclinic space group *Cm* (no. 8) has been identified from PXRD data (SI Tables S8, S11, S12) as apparent splitting of the main reflections has been observed after synthesis at different conditions. The *Cm* structure has been solved from PXRD data as no single crystals have been obtained.^[37]^ Energy dispersive X‐ray (EDX) spectroscopy of each sample has shown an atomic ratio *AE* : *Pn* : As of approximately 5 : 1 : 1 (SI Table S14) which matches the stoichiometry obtained by scXRD. O and N concentrations vary between the EDX datapoints due to the high air sensitivity of the samples and have therefore been neglected. The compounds feature a perovskite‐related structure, isostructural to PrBaCo_2_O_5_,^[31]^ where in our case the *A*‐site is occupied by pnictide elements, the *B*‐site by imide‐groups and the X‐site by alkaline earth elements and vacancies. The crystal structures consist of corner‐shared square‐pyramidal *AE*_5_NH units with imide groups pointing towards each other forming layers of vacancies (Figure 1a and d).

The *A*‐site is ordered showing alternating layers of As^3−^ and *Pn* ^3−^ occupations along the *c*‐axis, and as a result of the introduced vacancies two different coordination environments around the *A*‐site are formed. In the layer of vacancies a cube‐like *AE*_8_As coordination was observed (Figure 1c) alternating along the *c*‐axis by the regular icosahedral *AE*_12_(Sb/Bi) coordination (Figure 1b). As^3−^ always occupies the layers of vacancies, due to the smaller size of As^3−^ compared to Sb^3−^ and Bi^3−^ (Figure 1). Such an assumption can be derived from the series of Ca_3_ *Pn*N compounds. A distortion of the cubic perovskite structure (*Pm* $\bar{3}$ *m*) to an orthorhombic structure (*Pnma*) can be observed that has been similarly associated with the *Pn* ^3−^ radii sizes by several groups.[^19^, ^38^] Due to the alternating orientation of the imide‐groups and the introduced vacancies, two variants of *A*‐site coordination can be observed, namely *AE*_8_As‐cubes and *AE*_12_ *Pn*‐cuboctahedra.

### Pressure‐Dependent Polymorphs

**Figure 2:**
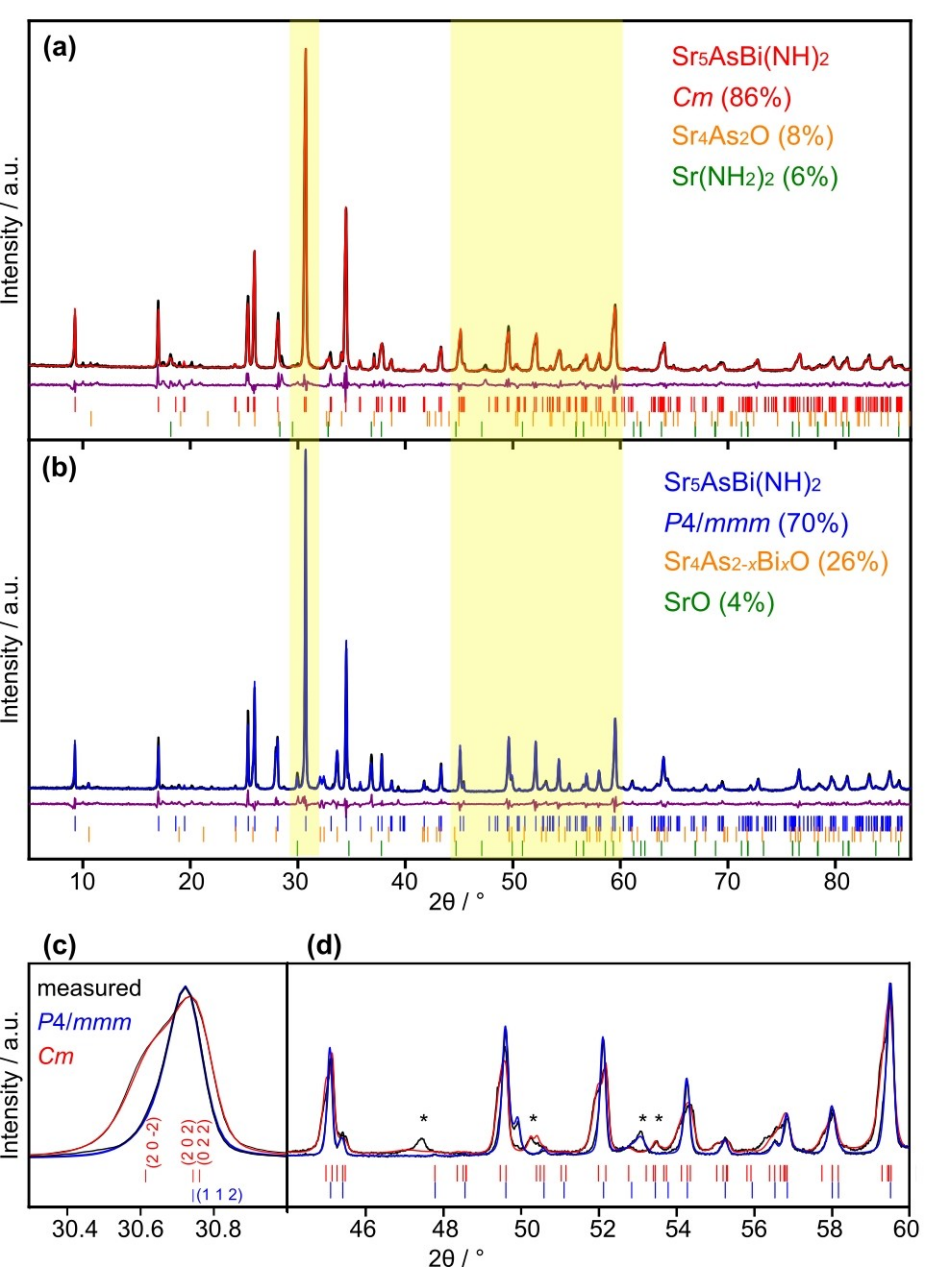
Rietveld refinement of Sr_5_AsBi(NH)_2_ (Cu‐$K_{\alpha 1}=1.540596$ Å), (a) monoclinic modification and (b) tetragonal modification. Experimental data are shown in black with the Rietveld fit in red (*Cm*) or blue (*P*4/*mmm*). Difference plot in purple with reflection positions of the corresponding phases marked by color coded streaks. (c) Comparison of the strongest reflex between the two modifications (d) Comparison of a selected area with reflexes of byproducts marked with asterisk.

All tetragonal modifications of *AE*_5_As*Pn*(NH)_2_ were synthesized at autogenous ammonia pressures of *p*>100 MPa. For Sr_5_AsBi(NH)_2_ a mixed occupation of the As^3−^‐site with Bi^3−^ was observed in the scXRD data, resulting in the composition Sr_5_As_0.88_Bi_1.12_(NH)_2_, which could stabilize the *P*4/*mmm* modification. However such a mixed occupation was not observed for Ca_5_AsSb(NH)_2_ and Ca_5_AsBi(NH)_2_.^[39]^ The monoclinic modifications were observed at lower pressures of *p*<80 MPa. Their lattice parameters show a √2 *a*_0_×√2 *a*_0_×*c* relation with a monoclinic angle of *β*=90.2−90.4°. By applying higher pressures during synthesis a *B*‐site ordering is introduced. The minor change of the symmetry was vaguely recognizable in the scXRD data. PXRD data in comparison show unambiguous evidence of the symmetry reduction with the lower symmetry modification featuring a splitting of reflections (Figure 2c and d).

Selected distances of the compounds in their *P*4/*mmm* modification are given in Table S9 (obtained from scXRD data). For the *Cm* modifications, distances were obtained from the pXRD data and are given in Table S10. The *AE*−*Pn* distances are all in the range of the *AE*−*Pn* distances found in the *AE*_3_ *Pn*N series, see Table S13 (*AE*=Ca, Sr; *Pn*=As, Sb, Bi).[^38^, ^40^] The *AE*_5_NH‐units in the title compounds with *P*4/*mmm* symmetry are composed of one short axial N(1)‐*AE*(1) distance and four long equatorial N(1)‐*AE*(2) distances. The NH^2−^ group in the *AE*_5_NH‐unit is shifted along the *c*‐axis, from an ideal octahedral coordination due to the introduced *AE*‐vacancies, which results in the observed short N(1)‐*AE*(1) distances (Ca_5_AsSb(NH)_2_: N(1)‐Ca(1)=2.27(4) Å; Ca_3_SbN: N−Ca=2.427 Å). Contrary, the equatorial N(1)‐*AE*(2) distances in the *P*4/*mmm* modification are slightly longer compared to N‐*AE* distances found in the corresponding *AE*_3_ *Pn*N compounds

(Ca_5_AsSb(NH)_2_/*P*4/*mmm*: N(1)‐Ca(2)=2.4462(6) Å; Ca_3_SbN: N−Ca=2.427 Å). The NH^2−^ group with the smaller charge and therefore smaller size compared to N^3−^ shows in the *Cm* modification a different coordination surrounding composed of three smaller N(1)‐*AE*(2) distances and two longer N(1)‐*AE*(3) distances (Ca_5_AsSb(NH)_2_/*Cm*: 1x N(1)‐*AE*(2)=2.35(8) Å; 2x N(1)‐*AE*(3)=2.33(4) Å; 2x N(1)‐*AE*(3)=2.58(5) Å) resulting in the reduction of the space group symmetry to *Cm*. Such a shift of the *B*‐site imide group away from the special position with 4*mm* site symmetry inside the *AE*_5_NH unit is typical for perovskite and related structures and often occurs in quaternary perovskite systems.[^41^, ^42^] The higher and lower symmetry modifications were observed for Ca_5_AsSb(NH)_2_ and Sr_5_AsBi(NH)_2_. Ca_5_AsBi(NH)_2_ has so far only been synthesized in the tetragonal modification

### NMR and Vibrational Spectroscopy

To further characterize the structure of the newly synthesized materials, vibrational spectroscopy as well as nuclear magnetic resonance spectroscopy was carried out. The ^1^H NMR spectra (see Figure S2) show two signals in all samples at around +1.8 and 0.7 ppm, which were assigned to the hydrogen atom in the NH^2−^ group. Compared to the reported ^1^H signal for CaNH at 5.3 ppm,^[43]^ a chemical shift to lower ppm values is expected due to the additional 4 As^3−^ ions in the local environment that are shielding the NH^2−^ group further. Additionally, with respect to a low amount of Ca(NH_2_)_2_ byproduct, we measured phase pure Ca(NH_2_)_2_ for comparison. A broad signal ranging from −3.0 to +0.7 ppm was observed and could be assigned to the detected signals at 0.7 ppm.

**Figure 3:**
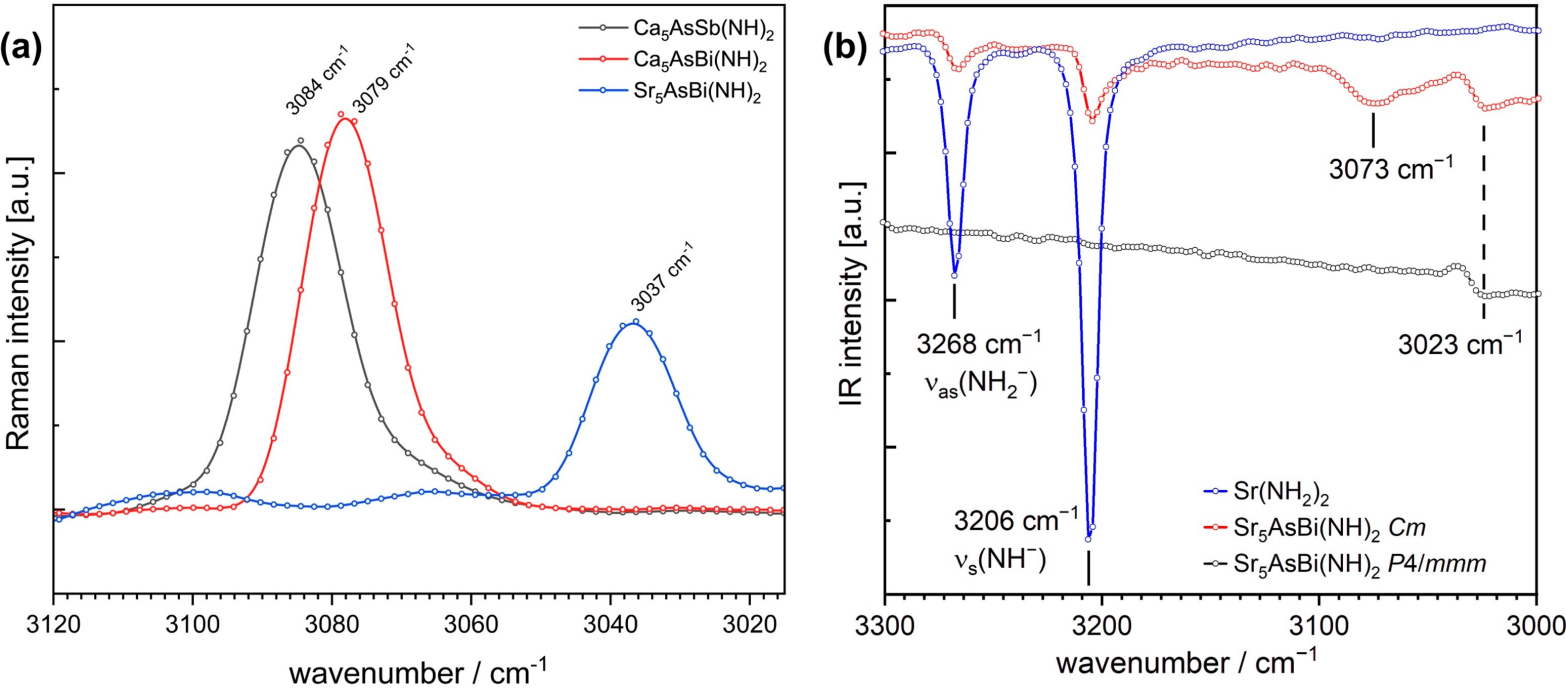
(a) Raman spectra of *AE*_5_As*Pn*(NH)_2_, (b) IR spectra of Sr_5_AsBi(NH)_2_ (*Cm* and *P*4/*mmm*) and Sr(NH_2_)_2_ (b). The region between 3000–3150 cm^−1^ typically shows the imide stretching vibration.[^43^, ^44^]

The presence of an imide‐group was further verified using vibrational spectroscopy (Figure 3 and Figure S3). IR‐spectroscopy measurements on the Cm samples show very weak signals at around 3073 cm^−1^ (Sr_5_AsBi(NH)_2_) which can be assigned to the stretching vibration of the NH^2−^ group, *ν*_s_(NH^2−^). In the case of the samples with *P*4/*mmm* symmetry no signal can be detected as the symmetry of the space group renders the vibration IR inactive. The very weak signal at 3023 cm^−1^ is most likely arising from an unknown byproduct. The impurity Sr(NH_2_)_2_ has been reported to show stretching vibrations at *ν*_as_(NH_2_)=3269 cm^−1^ and *ν*_s_(NH^−^)=3207 cm^−1^,^[44]^ which we were able to reproduce and sometimes observed depending on the sample quality. In Raman spectroscopy we observed the *ν*_s_(NH^2−^) vibration at 3037 cm^−1^ (Sr_5_AsBi(NH)_2_), at 3079 cm^−1^ (Ca_5_AsBi(NH)_2_), and at 3084 cm^−1^ (Ca_5_AsSb(NH)_2_), respectively. Similar shifts of the *ν*_s_(NH^2−^) vibration have been reported for the *AE*NH‐compounds (SrNH: 3183 cm^−1^; CaNH: 3143 cm^−1^) and can be correlated to the ionic radius of *AE* and subsequently increasing *AE*‐N distance.[^43^, ^44^] Furthermore, the IR spectra of Ca_5_AsSb(NH)_2_, Ca_5_AsBi(NH)_2_ and Sr_5_AsBi(NH)_2_ in *Cm* symmetry were calculated and are shown in Figure S6. Comparing the measured IR and Raman spectra with the simulated spectra, as shown in Figure S3, show an expected difference, where the imide stretching vibration in the region of 3000–3150 cm^−1^ is rendered inactive depending on the symmetry of the space group.

### Electronic Structure

**Figure 4:**
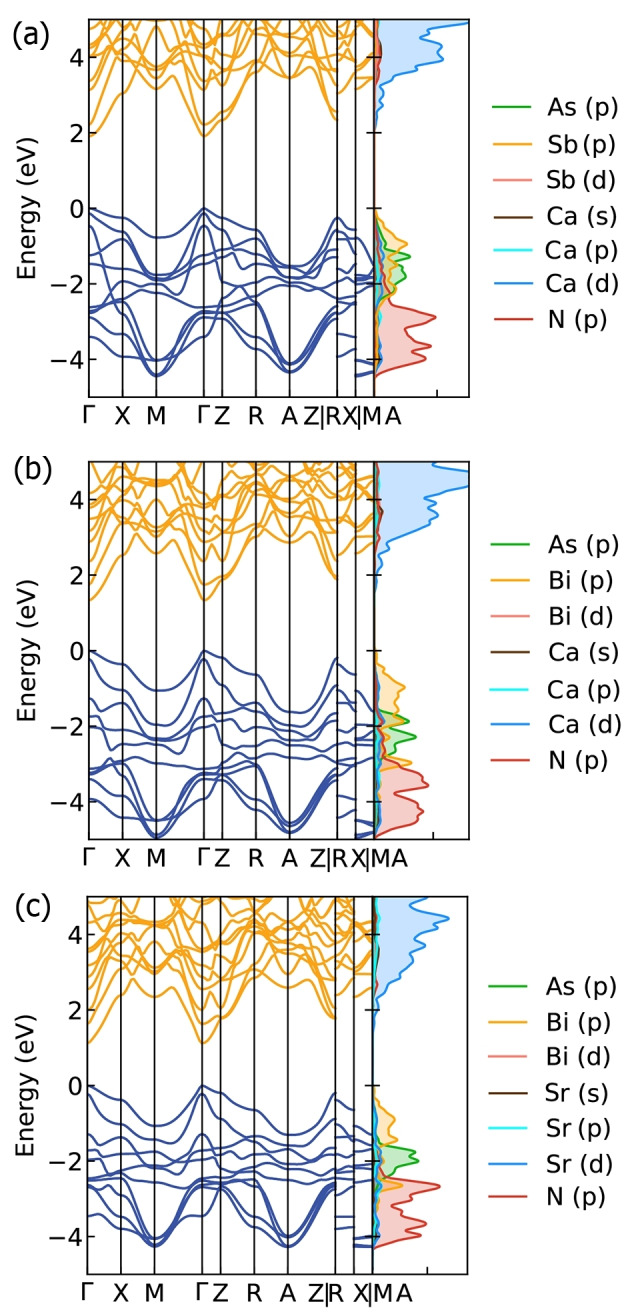
Band structure and density of states of (a) Ca_5_AsSb(NH)_2_, (b) Ca_5_AsBi(NH)_2_ and (c) Sr_5_AsBi(NH)_2_ in *P*4/*mmm* calculated at HSE+SOC level. The crystal structure with *P*4/*mmm* symmetry is used. The high symmetry k‐points in the first Brillouin Zone are Γ:(0,0,0), X:(0,0.5,0), M:(0.5,0.5,0), Z:(0,0,0.5), R:(0,0.5,0.5), A:(0.5,0.5,0.5).

Figure 4 exhibits the band structure and density of states (DOS) of Ca_5_AsSb(NH)_2_, Ca_5_AsBi(NH)_2_ and Sr_5_AsBi(NH)_2_ in *P*4/*mmm*. Those of Ca_5_AsSb(NH)_2_,and Sr_5_AsBi(NH)_2_ in *Cm* are displayed in Figure S4. All three compounds are semiconductors with direct band gaps at Γ point. The calculated band gaps of Ca_5_AsSb(NH)_2_, Ca_5_AsBi(NH)_2_ and Sr_5_AsBi(NH)_2_ in *P*4/*mmm* obtained using the hybrid functional (HSE) and accounting for the spin‐orbit coupling (SOC) are 1.91, 1.33 and 1.12 eV, respectively. Those of Ca_5_AsSb(NH)_2_ and Sr_5_AsBi(NH)_2_ in *Cm* are 1.93 and 1.14 eV, which exhibits a small difference with their counterparts in *P*4/*mmm*. Notably, the composition variation at the *X*‐site and *A*‐site can effectively tune the band gaps of these nitride antiperovskite derivatives. When Ca at the *X*‐site in Ca_5_AsBi(NH)_2_ is substituted with the heavier homologue Sr, the band gap decreases from 1.33 to 1.12 eV. On the other hand, as the Sb at the *A*‐site in Ca_5_AsSb(NH)_2_ is replaced by the heavier homologue Bi, the band gap decreases by 0.58 eV. The effective band gap tuning through the composition variation can be attributed to the direct contribution of the *X*‐site and *A*‐site elements to the electronic states at the band edges.

As displayed in Figure 4, the valence band minimum (VBM) is mainly composed of the *A*‐site elements Sb (Bi) p‐orbital while the conduction band maximum (CBM) is contributed by the *X*‐site elements Ca‐d orbital. Such a crossed band p‐d hybridization in combination with the enhanced covalency of pnictides lead to their large band dispersion,^[18]^ which would benefit carrier transport. Figure 1 shows that these new quaternary antiperovskite derivatives exhibit a two‐dimensional (2D) structure due to the interruption of the Ca−N octahedral network by substitution of two apical Ca atoms by H atoms. However, they exhibit three‐dimensional (3D) electronic properties, that is, besides the contribution of elements at the *X*‐site and *A*‐site to the band edge states, the *A*’‐site element As p‐orbital and the *B*‐site element N p‐orbital contribute to the upper valence band as well. 3D character of electronic transport would induce carrier mobility between two Ca_5_NH units via the As layer, and result in favorable out‐of‐plane transport properties of these novel defect imide double antiperovskites.

### Optical Properties

**Figure 5:**
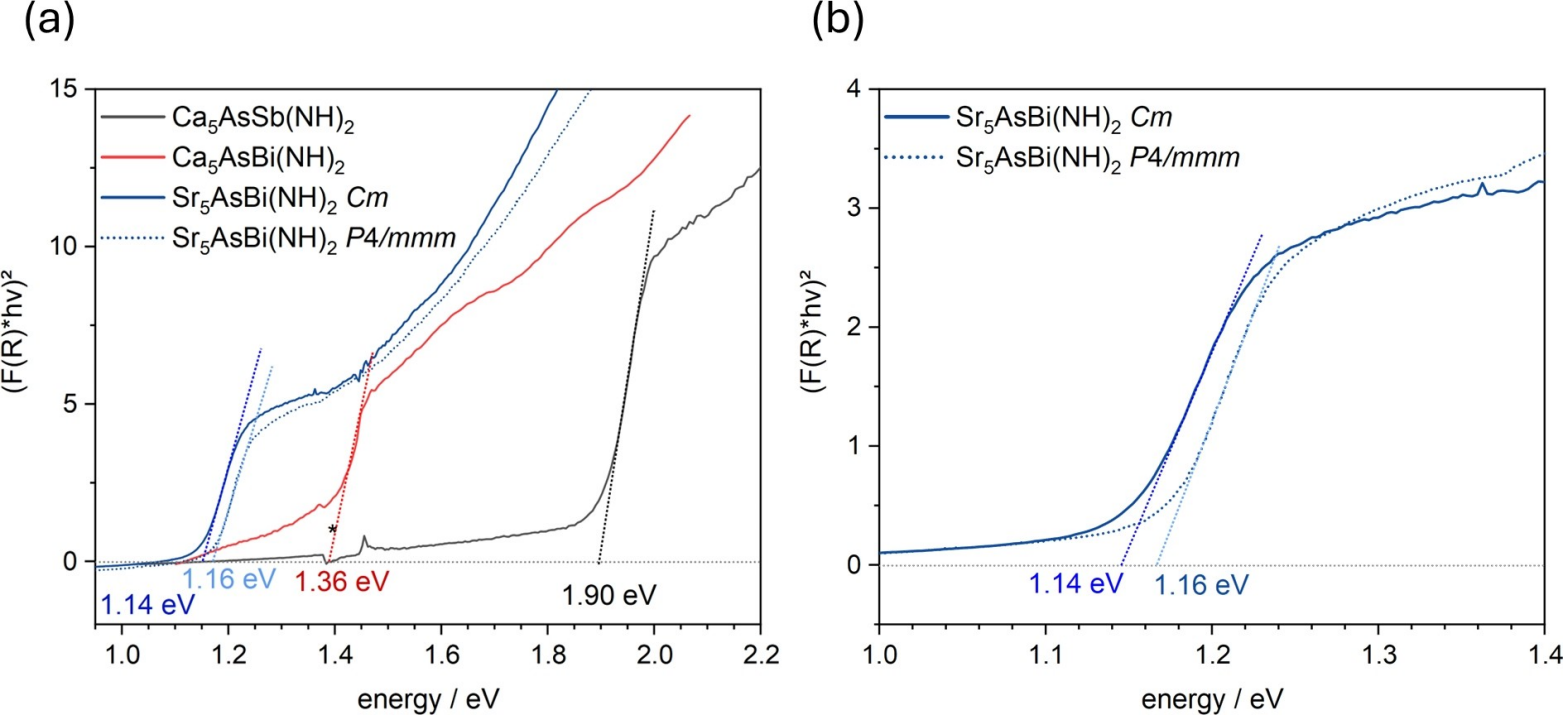
(a) TAUC‐plots of *AE*_5_As*Pn*(NH)_2_. Kubelka–Munk‐converted UV/Vis‐spectra of Ca_5_AsSb(NH)_2_ (black), Ca_5_AsBi(NH)_2_ (red), Sr_5_AsBi(NH)_2_ (*Cm*, solid blue) and Sr_5_AsBi(NH)_2_ (*P*4/*mmm*, dotted blue) are shown with extrapolation of the linear part for the determination of the optical band gaps. The signal marked with an asterisk originates from the setup. (b) TAUC‐plots of Sr_5_AsBi(NH)_2_ in *Cm* (no. 8) modification (solid) and *P*4/*mmm* modification (no. 123) (dotted).

To determine the optical band gap of *AE*_5_As*Pn*(NH)_2_ powder samples, diffuse reflectance UV/Vis spectroscopy was conducted. The obtained spectra were converted into tauc‐plots based on the Kubelka–Munk function. Band gap values were determined by extrapolating the absorption edges visible in the tauc‐plots with a linear fit function, see Figure 5. The experimentally obtained direct band gaps of Ca_5_AsSb(NH)_2_, Ca_5_AsBi(NH)_2_ and Sr_5_AsBi(NH)_2_ with *Eg*=1.90 eV, 1.36 eV and 1.14 eV, respectively, exhibit a shift to lower band gap energies from compounds comprising antimony to bismuth. Substituting the *AE* ^2+^ cation from Ca^2+^ to Sr^2+^ further leads to a band gap energy decrease of 0.22 eV. There is a good agreement between experimentally measured and theoretically calculated band gaps, both regarding the band gap values and the direct type of band gap (see Table 1 and Figure 7a). Comparing the band gaps of Sr_5_AsBi(NH)_2_ in its two different crystallographic modifications, namely the tetragonal modification in space group *P*4/*mmm* (no. 123) and the monoclinic modification in space group *Cm* (no. 8), it is notable that both band gaps with 1.16 eV (*P*4/*mmm*) and 1.14 eV (*Cm*) are almost identical and only differ by 0.02 eV, translating in an absorption‐onset difference of 18 nm.

**Table 1:** Comparison between calculated (at Γ point) and experimentally obtained band gap energies.

Formula space group	E_g_ (exp. direct)	E_g_ (calc. HSE‐SOC‐DOS)
Ca_5_AsSb(NH)_2_ *Cm*	1.90 eV	1.93 eV
Ca_5_AsBi(NH)_2_ *P*4/*mmm*	1.36 eV	1.33 eV
Sr_5_AsBi(NH)_2_ *P*4*/mmm* *Cm*	1.16 eV 1.14 eV	1.14 eV –

Furthermore, as shown in the calculated optical absorption spectrum of Sr_5_AsBi(NH)_2_ in *P*4/*mmm* and *Cm* (Figure S5), their slight difference in the out‐of‐plane absorption onsets may lead to the mild disparity of the experimental observation of the absorption onsets in the UV/Vis‐spectra. It can therefore be concluded that the band gap energies of the antiperovskite Sr_5_AsBi(NH)_2_ do not show a significant dependence on the crystallographic modification, and that both modifications are suitable materials for efficient light absorption as their band gaps reside within the desired energy range for favorable materials for single junction solar cells.

**Figure 6:**
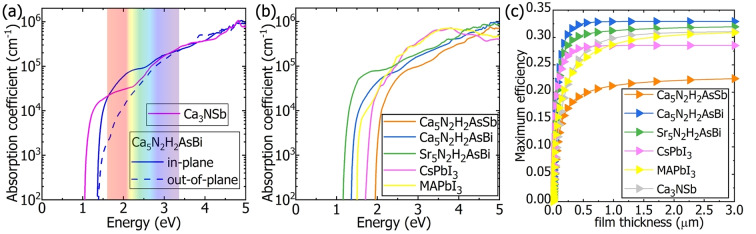
(a) The calculated absorption spectrum of Ca_5_AsBi(NH)_2_, and Ca_3_SbN; (b) absorption spectrum of averaged absorption coefficients of three newly‐synthesized multinary antiperovskite derivatives, and two representative halide perovskites; (c) SLME of the five compounds as shown in (b) as well as Ca_3_NSb. Ca_5_AsSb(NH)_2_ and Sr_5_AsBi(NH)_2_ in space group *Cm* and Ca_5_AsBi(NH)_2_ in space group *P*4/*mmm* are employed.

We take Ca_5_AsBi(NH)_2_ in *P*4/*mmm* as an example for the analysis of a theoretical absorption spectrum. As displayed in Figure 6a, the in‐plane and the out‐of‐plane absorption of Ca_5_AsBi(NH)_2_ onsets start at 1.33 eV, which is consistent with its fundamental band gap, reflecting the dipole‐allowed optical transition. Following the absorption onset, the in‐plane absorption coefficient sharply increases to 10^4^ cm^−1^ whilst the out‐of‐plane one slowly increases to 10^4^ cm^−1^ around 2 eV.

The suitable direct band gaps and dipole‐allowed optical transitions result in strong optical absorptions in the visible light range (1.65 to 3.10 eV) for these promising antiperovskite derivatives (Ca_5_AsSb(NH)_2_, Ca_5_AsBi(NH)_2_, and Sr_5_AsBi(NH)_2_), as shown in Figure 6b. The visible light absorption of Ca_5_AsBi(NH)_2_, and Sr_5_AsBi(NH)_2_ is comparable to that of CsPbI_3_ and MAPbI_3_. Furthermore, the spectroscopic limited maximum efficiency (SLME) of the three compounds and two halide perovskites as function of the film thickness was calculated. The calculated maximum efficiency of a MAPbI_3_ based solar cell with a 3 μm‐thick film is 30.90 % as shown in Figure 6c, which is in agreement with a preceding calculated efficiency.^[45]^ All of the studied compounds exhibit a sharp increase of efficiency at a film thickness of 0.3 μm, and in such a very thin absorber layer, a conversion efficiency of 18.20 %, 31.64 %, and 29.54 % can be achieved for Ca_5_AsSb(NH)_2_, Ca_5_AsBi(NH)_2_, and Sr_5_AsBi(NH)_2_, respectively, as shown in Figure 6c. The SLME of these compounds is predicted to reach values as high as 22.49 %, 32.93 %, and 31.97 % when the thickness of the film increases to 3.0 μm, respectively. Remarkably, the SLMEs of the latter (Ca_5_N_2_H_2_AsBi and Sr_5_N_2_H_2_AsBi) are higher than those of MAPbI_3_ (30.90 %), CsPbI_3_ (28.55 %) and Ca_3_NSb (31.06 %).

### Transport Properties

**Figure 7:**
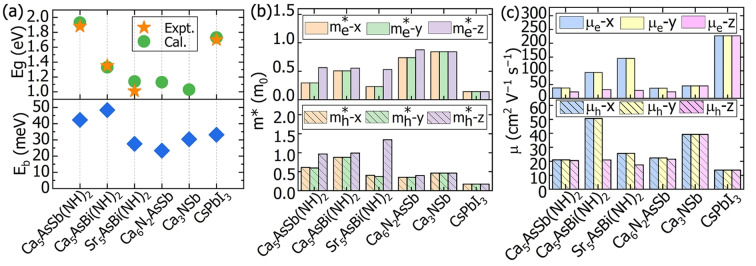
(a) Band gaps (*E*_g_) and calculated exciton binding energies (*E*_b_), (b) computed effective masses and (c) mobilities of three newly synthesized multinary antiperovskite derivatives, two nitride antiperovskites and one representative halide perovskite. Ca_5_AsSb(NH)_2_ and Sr_5_AsBi(NH)_2_ in space group *Cm* and Ca_5_AsBi(NH)_2_ in space group *P*4/*mmm* are employed.

As shown in the bottom panel of Figure 7a, the theoretically estimated Wannier‐Mott exciton binding energies (*E*_b_) of Ca_5_AsSb(NH)_2_, Ca_5_AsBi(NH)_2_, and Sr_5_AsBi(NH)_2_ are 42, 48 and 28 meV, respectively, which are comparable and even smaller than that of MAPbI_3_ with 45 meV calculated by the same method.^[46]^ Figure 7b shows that the effective masses of these multinary antiperovskite derivatives are anisotropic. The electron effective masses (m*_e_) along three directions are much smaller than the rest mass of the electron m_0_. The in‐plane hole effective masses (m*_h_) are less than m_0_ while the out‐of‐plane ones are within the range of 0.9–1.6 m_0_. Notably, the in‐plane hole effective masses are comparable to those of 3D nitride antiperovskites, whilst the out‐of‐plane ones are 2–3 times larger than that of Ca_3_NSb, as shown in Figure 7b. Furthermore, the carrier mobilities were calculated at room temperature and a doping level of 10^16^ cm^−3^ to straightforwardly evaluate the transport properties. The experimentally measured overall mobilities of CsPbI_3_ by different methods are within the range of 1.5–300 cm^2^ V^−1^ s^−1^.[^47^, ^48^, ^49^, ^50^]

Our calculated mobilities of CsPbI_3_ are also within this range.^[51]^ The hole and electron mobilities of our three multinary antiperovskite derivatives are within the range of 12–145 cm^2^ V^−1^ s^−1^. Among these compounds, Ca_5_AsBi(NH)_2_ and Sr_5_AsBi(NH)_2_ present the largest in‐plane hole and electron mobilities, while Ca_5_AsSb(NH)_2_ and Sr_5_AsBi(NH)_2_ feature the highest out‐of‐plane hole and electron mobilities, which are on par with 3D antiperovskites Ca_6_AsSbN_2_ and Ca_3_NSb, as displayed in Figure 7c. The 3D character of electronic transport facilitates the enhanced out‐of‐plane transport properties of the three newly‐synthesized multinary antiperovskite derivatives.

Owing to high air‐sensitivity and the challenging thin film preparation, the experimental characterization of mobilities has not been carried out yet and is subject of ongoing research.

## Conclusion

To conclude, we synthesized and characterized a series of novel defect antiperovskite‐related compounds

*AE*_5_As*Pn*(NH)_2_ (*AE*=Ca, Sr; *Pn*=Sb, Bi). The series of compounds shows band gaps in the range of 1.14–1.90 eV and under inert conditions, they remain stable upon exposure to heat (up to 1073 K) and light. Theoretical calculations suggest high electron and hole mobilities as well as suitable absorption spectra leading to calculated maximum efficiencies of up to 31.97 %, making them comparable to MAPbI_3_, the current gold standard for perovskite‐based solar cells. These properties are desirable for future device applications. However, current challenges for most antiperovskite‐based materials include handling of the air‐ and moisture‐sensitive compounds as well as in situ film preparation. The newly discovered compounds would require a thin film preparation of antiperovskite materials using ammonothermal conditions, which remains challenging due to the demanding nature of these conditions for existing substrate materials. The high thermal stability, however, could render the compounds suitable for e‐beam deposition or as sputter targets for thin‐film preparations. This work presents a distinct approach for designing new lead‐free perovskite‐based photovoltaic absorber materials and offer a path towards new unexplored perovskite‐structured nitrides.

## Supporting Information

### Supplementary Methods

Additional experimental details, Detailed single crystal data, Detailed Rietveld refinement data, Selected distances in *AE*_5_As*Pn*(NH)_2_, ^1^H MAS‐NMR of *AE*_5_As*Pn*(NH)_2_, IR‐spectra of Sr_5_AsBi(NH)_2_ in *Cm* vs. *P*4/*mmm*, EDX‐data.

### Supplementary Calculations

Convergence test of mobilities, IR and Raman calculation, ERGRE (DOC).

## Conflict of Interests

The authors declare no conflict of interest.

1

## Supporting information

As a service to our authors and readers, this journal provides supporting information supplied by the authors. Such materials are peer reviewed and may be re‐organized for online delivery, but are not copy‐edited or typeset. Technical support issues arising from supporting information (other than missing files) should be addressed to the authors.

Supporting Information

## Data Availability

The data that support the findings of this study are available in the supplementary material of this article.
