# Supplementary material for: Defect Imide Double Antiperovskites AE 5AsPn(NH)2 (AE=Ca, Sr; Pn=Sb, Bi) as Potential Solar Cell Absorber Materials
Source: Angew Chem Int Ed Engl. 2025 Feb 25;64(17):e202500768. doi: 10.1002/anie.202500768 (PMC12015382; doi:10.1002/anie.202500768)
Supplement: Supplementary file 1 — Supporting Information [file ANIE-64-e202500768-s001.pdf]

Angewandte Chemie
Eine Zeitschrift der Gesellschaft Deutscher Chemiker

Supporting Information

Defect Imide Double Antiperovskites $AE_5AsPn(NH)_2$ (AE = Ca, Sr; Pn = Sb, Bi) as Potential Solar Cell Absorber Materials

*T. G. Chau, D. Han, F. Wolf, S. S. Rudel, Y. Yao, H. Oberhofer, T. Bein, H. Ebert, W. Schnick**

Supporting Information

Table of contents

1. **Supplementary Methods**
   - **1.1 Experimental Details**
   - **1.2 Detailed single crystal data**
   - **1.3 Detailed Rietveld refinement data**
   - **1.4 ^{1}H MAS NMR of *AE*$_5$As*Pn*(NH)$_2$**
   - **1.5 IR-Spectra of $Sr_5AsBi(NH)_2$ (*Cm* vs *P*4/*mmm*)**
   - **1.6 EDX**
2. **Supplementary calculation**
   - **2.1 Convergence test of mobilities**
   - **2.2. Electronic structure of $Ca_5AsSb(NH)_2$ and (b) $Sr_5AsBi(NH)_2$ in *Cm*.**
   - **2.3. Calculated optical spectra of $Sr_5AsBi(NH)_2$ (*Cm* vs *P*4/*mmm*)**
   - **2.4 IR spectrum of *AE*$_5$As*Pn*(NH)$_2$ calculated and experimental**

1. Supplementary Methods

1.1 Experimental Details

Reagents

AsSb was prepared using a stoichiometric mixture of As (Alfa Aesar, 99.9 %, 3746.1 mg, 50 mmol) and Sb (Riedel-de Haen, 99 %, 6088.0 mg, 50 mmol). The reaction was carried out using a planetary ball mill (PM 200, Retsch) with the following parameters: 48 h reaction time, 500 rpm, 5 min grinding time, 5 min break, 10 ZrO_2:Y grinding balls (size 10 mm), 50 ml ZrO_2:Y grinding jar. CaH_2 (Sigma Aldrich, 99.99 %), SrH_2 (Materion, 99.5 %), As (Alfa Aeser, 99.9 %), and Bi (Merck, 99 %) were used as supplied. NH_3 (5.0, Air liquide) was purified with a MicroTorr MC400 702FV cartridge (SAES Pure Gas, Inc.).

All samples were handled inside a glovebox under argon atmosphere due to the high air-sensitivity of the samples (*c*(O2) < 0.1 ppm, *c*(H2O) < 1 ppm, UNIlab, MBRAUN, Garching, Germany).

Synthesis

AE$_5$As*Pn*(NH)$_2$ compounds were synthesized from CaH_2, or SrH_2 (Materion, 99.5 %), AsSb, As or Bi, as required, in an ammonothermal reaction. Custom-made ammonothermal autoclaves (autoclave volume: 10 ml, Haynes 282) equipped with niobium liners were used as reaction vessels. The starting materials were ground in an agate mortar and placed in the autoclave within an open liner. Ammonia was condensed into the autoclave via condensation by a N_2/Ethanol mixture. The autoclave was heated using a three-zone oven for an increased control of the temperature gradient. Subsequently, the autoclave was cooled to room temperature and the residual ammonia was removed.[1]

$Ca_5AsSb(NH)_2$ was obtained in two modifications with *Cm* and *P*4/*mmm* symmetry, respectively. The *Cm* modification was synthesized from CaH_2 (7.5 mmol, 315.8 mg) and AsSb (1.5 mmol, 295.0 mg). The autoclave was heated to 400 °C within 2 h and kept at this temperature for 12 h before the temperature was raised to 800°C within 4 h and held for 30 h. An autogenous pressure of 78 MPa was reached after the second heating step. For the *P*4/*mmm* modification of $Ca_5AsSb(NH)_2$, CaH_2 (4.5 mmol, 189.5 mg) and AsSb (0.75 mmol, 147.5 mg) was used with additional KN_3 (1.0 mmol, 81.1 mg) as a mineralizer with the intention to further promote the growth of larger single crystals. The autoclave for the *P*4/*mmm* modification synthesis was heated to 400 °C within 2 h and kept at this temperature for 12 h before the temperature was raised to 750 °C within 4 h and held for 30 h. An autogenous maximum pressure of 110 MPa was reached after the second heating step.

$Ca_5AsBi(NH)_2$ was obtained only in the tetragonal *P*4/*mmm* modifications. It was synthesized using CaH_2 (8.25 mmol, 347.3 mg), As (1.5 mmol, 112.4 mg) and Bi (1.5 mmol, 313.5 mg). The autoclave was heated to 400 °C within 2 h and kept at this temperature for 12 h before the temperature was raised to 750 °C within 4 h and held for 30 h. An autogenous pressure of 110 MPa was reached after the second heating step. An additional annealing step under ammonothermal conditions was included for which the samples were ground again, heated to 400 °C within 2 h and kept at this temperature for 12 h before the temperature was raised to 750 °C within 4 h and held for 20 h. During the additional annealing step, an autogenous maximum pressure of 90 MPa was reached.

$Sr_5AsBi(NH)_2$ was obtained in the two modifications *Cm* and *P*4/*mmm.* The *Cm* modification was synthesized from SrH_2 (6.0 mmol, 537.7 mg) and a ball-milled As and Bi mixture (1:1, in total 283.9 mg). The autoclave was heated to 400 °C within 2 h and kept at this temperature for 12 h before the temperature was raised to 750 °C within 4 h and held for 30 h. An autogenous maximum pressure of 50 MPa was reached after the second heating step. For the *P*4/*mmm* modification of $Sr_5AsBi(NH)_2$ SrH_2 (5.0 mmol, 448.1 mg) and the As and Bi mixture (1:1, in total 283.9 mg) was used. For the *P*4/*mmm* modification synthesis, the autoclave was heated to 400 °C within 2 h and kept at this temperature for 12 h before the temperature was raised to 800 °C within 4 h and held for 40 h. An autogenous maximum pressure of 120 MPa was reached after the second heating step.

Single crystal X-ray diffraction

Single crystal X-ray diffraction data of $Ca_5AsBi(NH)_2$ were collected using a Bruker D8 Quest. Single crystal X-ray diffraction data of $Ca_5AsSb(NH)_2$ and $Sr_5AsBi(NH)_2$ were collected using a Bruker D8 Venture. All crystals were prepared in glass capillaries (Hilgenberg GmbH, Germany) under dried paraffin oil. For the data collection, indexing, data reduction and absorption correction (multi scan) APEX 3 software[2] was used. The XPREP[3] software was used for the analysis of systematically absent reflections and space group determination. Structure solutions were carried out in WINGX[4] using SHELXT.[5] For the refinement the SHELXLE software[6] was used. The H-atoms were fixed and refined isotropically at the highest residual electron densities in the vicinity of N-atoms. For single crystals crystallizing in the monoclinic structure (*Cm*) no reliable structure solution and refinement was achieved due the tetragonal arrangement of the heavy atoms (*AE*, Sb, Bi) and high degree of twinning. However, a *Cm*-structure model was obtained which was used for the following Rietveld refinement from powder X-ray diffraction data.

Single crystal X-ray diffraction data of $Ca_5AsSb(NH)_2$, $Ca_5AsBi(NH)_2$ and $Sr_5AsBi(NH)_2$ in *P*4/*mmm* and the monoclinic structures in *Cm* obtained from powder X-ray diffraction data were deposited under CSD number 2412540-2412544. These data are provided free of charge by the joint Cambridge Crystallographic Data Centre and Fachinformationszentrum Karlsruhe Access Structures service www.ccdc.cam.ac.uk/structures.

Powder X-ray diffraction

Powder X-ray diffraction data were collected using a STOE StadiP diffractometer equipped with Cu-$K_{\alpha1}$ (λ = 1.5406 Å) radiation, a Ge(111) monochromator and a DECTRIS MYTHEN 1K Si-strip detector in modified Debye-Scherrer geometry. Samples were sealed in glass capillaries (special glass no.10, Hilgenberg GmbH, Germany). Due to the high absorption all samples were diluted with amorphous Boron (Sigma Aldrich, ≥ 95 %). Topas6[7] was used with a fundamental parameters refinement approach for the Rietveld refinement of the obtained data. All atoms were refined isotropically and the background was described using the Shifted Chebyshev polynomial with 10 parameters.

IR and Raman Spectroscopy

Raman measurements were conducted on a Bruker Vertex 70 spectrometer with a Ram II FTIR/Raman extension equipped with a Coherent 1064 nm laser with a maximum power of 500 mW and a liquid nitrogen cooled IN-Ge NIR-detector. Spectra were recorded in reflectance with the laser defocused and with different laser intensities between 10 mW and 100 mW to prevent thermal degradation of the sample. Air-sensitive samples were encapsulated inside a N_2-filled glove box between two transparent glass plates using a solvent-free two-component adhesive (Torr Seal Epoxy Resin, Agilent Technologies, Inc., USA). Spectra were baseline-corrected, using the “Concave Rubberband Correction” method. IR measurements were conducted on a Bruker Alpha II FTIR spectrometer using a Platinum-ATR unit equipped with a diamond crystal.. All spectra were recorded in a glovebox in the range of 400–4000cm^{-1}.

UV/Vis Spectroscopy

UV/Vis measurements were performed on a Perkin Elmer Lambda 1050 UV/VIS/NIR spectrophotometer equipped with a 150 mm integrating sphere and a custom-built spot reduction kit. Air-sensitive samples were encapsulated inside a N_2-filled glove box between two transparent glass plates using a solvent-free two-component adhesive (Torr Seal Epoxy Resin, Agilent Technologies, Inc., USA). Spectra were recorded in reflectance. TAUC-Plots were calculated using the Kubelka-Munk function.[8]

SEM/EDX Spectroscopy

Direct SEM image preparation was done using an FEI Helios Nanolab G3 UC, equipped with a Schottky-type field-emitter, operated between 1 and 30 kV. All measurements showed a high degree of oxygen content, which should not be taken into account as the sample is exposed to air for the measurement preparation and consequently hydrolyzes.

Computational Methods

First-principles calculations were carried out using the Vienna Ab initios Simulation package (VASP).[9] The generalized gradient approximation (GGA) in the Perdew-Burke-Ernzerhof (PBE) form was employed for the exchange-correlation functional.[10] The kinetic-energy cutoff was chosen as 520 eV. The convergence criteria for the energy and forces were set to 10^{-5} eV and 0.01 eV/Å, respectively. For the Brillouin zone integration, the Monkhorst-Pack k-point mesh with a grid spacing of ~2πx0.03 eV/Å was used. A Hybrid functional (HSE06) with 40% non-local Fock exchange was employed for the electronic structure calculations.[11] Spin-orbit coupling was taken into consideration for the compounds with heavy p-electron elements like Sb and Bi.[12,13] The software SUMO has been utilized for the postprocessing of band structures, density of states and effective mass.[14] The Wannier-Mott exciton binding energy (Eb) was estimated using a modified hydrogen-atom-like Bohr model.[15] A dense k-point mesh (less than 2πx0.01 eV/Å) was employed for the calculation of optical absorption spectra. The theoretical conversion efficiency of the solar cells namely SLME was computed based on the method proposed by Yu, L. et al. and a python code (SL3ME).[16]

The electrical transport properties of $Ca_5AsSb(NH)_2$, $Ca_5AsBi(NH)_2$ and $Sr_5AsBi(NH)_2$ were obtained via the momentum relaxation time approximation (MRTA) as implemented in AMSET under p-type and n-type doping conditions with carrier concentrations in the order of 10^{16} cm^{-3}.[17] Three scattering processes namely the acoustic deformation potential (ADP), ionized impurity (IMP), and polar-optical phonon (POP) scattering were considered. The material parameters including high-frequency and static dielectric constants, elastic constants, polar optical phonon frequency and deformation potentials were all obtained via first-principles calculations. The dielectric constants, and the "effective polar optical phonon frequency" were obtained using density-functional perturbation theory (DFPT) using the exchange-correlation functional.[18] Elastic constants were calculated through the stress-strain approach also using the PBE functional. The transport properties need to be converged with respect to an interpolation factor which controls the number of k-points in the interpolated band structures. Convergence testing results of $Ca_5AsSb(NH)_2$ are provided in Table S15. A dense Fourier interpolated mesh of 97x97x53 was chosen (the original input k-point meshes of the electronic band structures computed using DFT were 18x18x10) for $Ca_5AsSb(NH)_2$ and $Sr_5AsBi(NH)_2$ in *Cm* and 65x65x37 (12x12x7 DFT mesh) for $Ca_5AsBi(NH)_2$ in *P*4/*mmm*. The convergence criteria for energy (10^{-8} eV) and forces (0.001 eV/Å) were utilized for the geometry optimization of the crystal structures, and the same tight settings were used for the calculations of electrical transport properties.

For the theoretical IR and Raman spectra, the vibrational properties were calculated making use of the FHI-aims all electron code, based on numerically tabulated atom-centered orbitals (NAOs) using the finite difference approach, with a finite displacement δ of 0.002 Å.[19] The response of the electric field to the displacement was computed with density-functional perturbation theory (DFPT).[20] Standard "light" computational settings (basis set and integration grids) were employed and exchange and correlation were treated with the GGA PBE functional.[10,21] Note that light settings in FHI-aims are comparable to a split valence polarized triple zeta basis in more common Gaussian basis codes.[22]

1.2 Detailed Single Crystal data

Table S1: Detailed data of the single crystal structure refinement of $Ca_5AsSb(NH)_2$, $Ca_5AsBi(NH)_2$ and $Sr_5As_{0.88}Bi_{1.12}(NH)_2$.

formula	$Ca_5AsSb(NH)_2$	$Ca_5AsBi(NH)_2$	$Sr_5As_{0.88}Bi_{1.12}(NH)_2$
crystal system		tetragonal	
space group		*P*4/*mmm* (no.123)	
formula weight / g *mol^{-1}	427.11	514.34	752.04
a / Å	4.8685(3)	4.8756(8)	5.1906(2)
c / Å	8.9249(6)	8.9317(14)	9.5118(3)
V / $Å^3$	211.54(3)	212.32(8)	256.27(2)
Z	1	1	1
Diffractometer		Bruker D8 Venture	
Radiation type		$Mo_{K\alpha}$ (0.71074 nm)	
Temperature / K		293	
Goodness of fit (Goof)	1.328	1.180	1.269
µ / mm^{-1}	10.083	27.558	47.735
Calculated X-ray density / g·cm^{-3}	3.353	4.023	4.977
F(000)	200	232	322
θ range / °	4.186 < 2θ <33.123	4.180 < 2θ <34.958	3.926< 2θ <33.128
Total no. of reflections	4055	8760	9688
Independent reflections [I≥2σ(I) / all]	288	319	340
R_1 ($F^2 \geq 2\sigma(F^2)$ / all)	0.019 / 0.021	0.017 / 0.018	0.013 / 0.014
wR_2 ($F^2 \geq 2\sigma(F^2)$ / all)	0.041 / 0.042	0.039 / 0.039	0.025 / 0.026
R_{int} / R_σ	0.028 / 0.017	0.060 / 0.017	0.040/ 0.019
$\Delta\rho_{max}$; $\Delta\rho_{min}$ / e*$Å^{-3}$	1.114; –0.673	1.081; –1.890	1.178;-1.041

Table S2: Atomic coordinates, Occupancy, Wyckoff symbol and equivalent displacement parameters ($Å^2$) of $Ca_5AsSb(NH)_2$ from single crystal data.

Atom	Wyckoff	*x*	*y*	*z*	U_{eq}	s.o.f
Sb01	1*b*	1	1	1/2	0.01009(13)	1
As02	1*a*	0	0	0	0.00631(16)	1
Ca03	4*i*	0	1/2	0.21526(8)	0.01109(14)	1
Ca04	1*d*	1/2	1/2	1/2	0.0127(3)	1
N005	2*h*	1/2	1/2	0.2422(6)	0.0079(7)	1
H006	2*h*	1/2	1/2	0.160(11)	0.03(3)	1

Table S3: Anisotropic displacement parameters (Å^2) of $Ca_5AsSb(NH)_2$ from single-crystal data.

Atom	U_{11}	U_{22}	U_{33}	U_{12}	U_{13}	U_{23}
Sb01	0.0101(2)	0.0101(2)	0.0105(2)	0.00000	0.00000	0.00000
As02	0.0067(2)	0.0067(2)	0.0057(3)	0.00000	0.00000	0.00000
Ca03	0.0069(3)	0.0116(3)	0.0150(3)	0.00000	0.00000	0.00000
Ca04	0.0162(5)	0.0162(5)	0.0058(6)	0.00000	0.00000	0.00000
N005	0.0074(10)	0.0074(10)	0.0088(16)	0.00000	0.00000	0.00000

Table S4: Atomic coordinates, Occupancy, Wyckoff symbol and equivalent displacement parameters (Å^2) of $Ca_5AsBi(NH)_2$ from single crystal data.

Atom	Wyckoff	*x*	*y*	*z*	U_{eq}	s.o.f
Bi1	1*b*	1	1	1/2	0.01137(9)	1
As1	1*a*	0	0	0	0.0082(2)	1
Ca1	1*d*	1/2	1/2	1/2	0.0123(3)	1
Ca2	4*i*	1/2	0	0.78675(11)	0.0117(2)	1
N005	2*h*	1/2	1/2	0.7575(6)	0.0087(8)	1
H006	2*h*	1/2	1/2	0.86230	0.073	1

Table S5: Anisotropic displacement parameters (Å^2) of $Ca_5AsBi(NH)_2$ from single-crystal data.

Atom	U_{11}	U_{22}	U_{33}	U_{12}	U_{13}	U_{23}
Bi1	0.01187(11)	0.01187(11)	0.01035(15)	0.00000	0.00000	0.00000
As1	0.0085(2)	0.0085(2)	0.0076(3)	0.00000	0.00000	0.00000
Ca1	0.0152(5)	0.0152(5)	0.0064(6)	0.00000	0.00000	0.00000
Ca2	0.0122(4)	0.0079(4)	0.0151(4)	0.00000	0.00000	0.00000
N5	0.0089(12)	0.0089(12)	0.008(2)	0.00000	0.00000	0.00000

Table S6: Atomic coordinates, Occupancy, Wyckoff symbol and equivalent displacement parameters (Å^2) of $Sr_5As_{0.88}Bi_{1.12}(NH)_2$ from single crystal data.

Atom	Wyckoff	*x*	*y*	*z*	U_{eq}	s.o.f
As01	1*a*	0	0	0	0.0106(2)	0.879
Bi01	1*a*	0	0	0	0.0106(2)	0.121
Bi02	1*b*	1	1	1/2	0.01276(7)	1
Sr03	4*i*	0	1/2	0.21199(4)	0.01536(8)	1
Sr04	1*d*	1/2	1/2	1/2	0.01652(13)	1
N1	2*h*	1/2	1/2	0.2433(5)	0.0108(6)	1
H1	2*h*	1/2	1/2	0.133(2)	0.11(5)	1

Table S7: Anisotropic displacement parameters (Å^2) of $Sr_5As_{0.88}Bi_{1.12}(NH)_2$ from single-crystal data.

Atom	U_{11}	U_{22}	U_{33}	U_{12}	U_{13}	U_{23}
As01	0.0106(2)	0.0106(2)	0.0106(3)	0.00000	0.00000	0.00000
Bi01	0.0106(2)	0.0106(2)	0.0106(3)	0.00000	0.00000	0.00000
Bi02	0.01233(9)	0.01233(9)	0.01363(12)	0.00000	0.00000	0.00000
Sr03	0.01008(14)	0.01497(15)	0.02104(15)	0.00000	0.00000	0.00000
Sr04	0.0196(2)	0.0196(2)	0.0104(3)	0.00000	0.00000	0.00000
N1	0.0103(9)	0.0103(9)	0.0118(15)	0.00000	0.00000	0.00000

1.3 Detailed Rietveld refinement data

Table S8: Detailed Rietveld refinement data of $Ca_5AsSb(NH)_2$ (*P*4/*mmm* and *Cm*), $Sr_5AsBi(NH)_2$ (*P*4/*mmm* and *Cm*) and $Ca_5AsBi(NH)_2$ (*P*4/*mmm*)

**formula**	$\mathbf{Ca_5AsSb(NH)_2}$	$\mathbf{Ca_5AsBi(NH)_2}$	$\mathbf{Sr_5As_{0.89}Bi_{1.11}(NH)_2}$	$\mathbf{Ca_5AsSb(NH)_2}$	$\mathbf{Sr_5AsBi(NH)_2}$
crystal system	tetragonal	tetragonal	tetragonal	monoclinic	monoclinic
space group	*P*4/*mmm* (no. 123)	*P*4/*mmm* (no. 123)	*P*4/*mmm* (no. 123)	*Cm* (no.8)	*Cm* (no.8)
formula weight / g *mol^{-1}	427.11	514.34	752.04	427.11	752.04
a / Å	4.86863(6)	4.88326(4)	5.19228(6)	6.9115(6)	7.3557(6)
b / Å	4.86863(6)	4.88326(4)	5.19228(6)	6.8944(5)	7.3347(4)
c / Å	8.92710(15)	8.94970(11)	9.50698(18)	8.9635(9)	9.5018(10)
β / °	90	90	90	90.375(8)	90.243(6)
V / Å^3	211.605(7)	213.417(5)	256.307(8)	427.11(6)	512.64(7)
Z	1	1	1	2	2
Diffractometer			Stoe STADI P		
Radiation type			Cu-$K_{\alpha1}$ (1.54060 Å)		
Monochromator			Ge(111)		
Detector			Mythen 1K		
Background function			shifted Chebyshev, 10 polynomials		
Calculated X-ray density / g·cm^{-3}	3.3516(1)	4.0018(1)	4.950(2)	3.191(9)	4.8588(7)
θ range / °	$5 < 2\theta < 101.9$	$5 < 2\theta < 100.5$	$5 < 2\theta < 102.6$	$5 < 2\theta < 100.1$	$5 < 2\theta < 105.3$
Data points	6600	6372	6641	6468	6685
Total no. of reflections	96	93	118	244	329
Refined parameters	43	43	50	51	50
Goodness of fit (Goof)	1.425	1.137	1.455	1.266	1.439
R_p	0.077	0.067	0.067	0.062	0.071
R_{wp}	0.096	0.090	0.090	0.081	0.098
R_{Bragg}	0.070	0.028	0.025	0.037	0.035

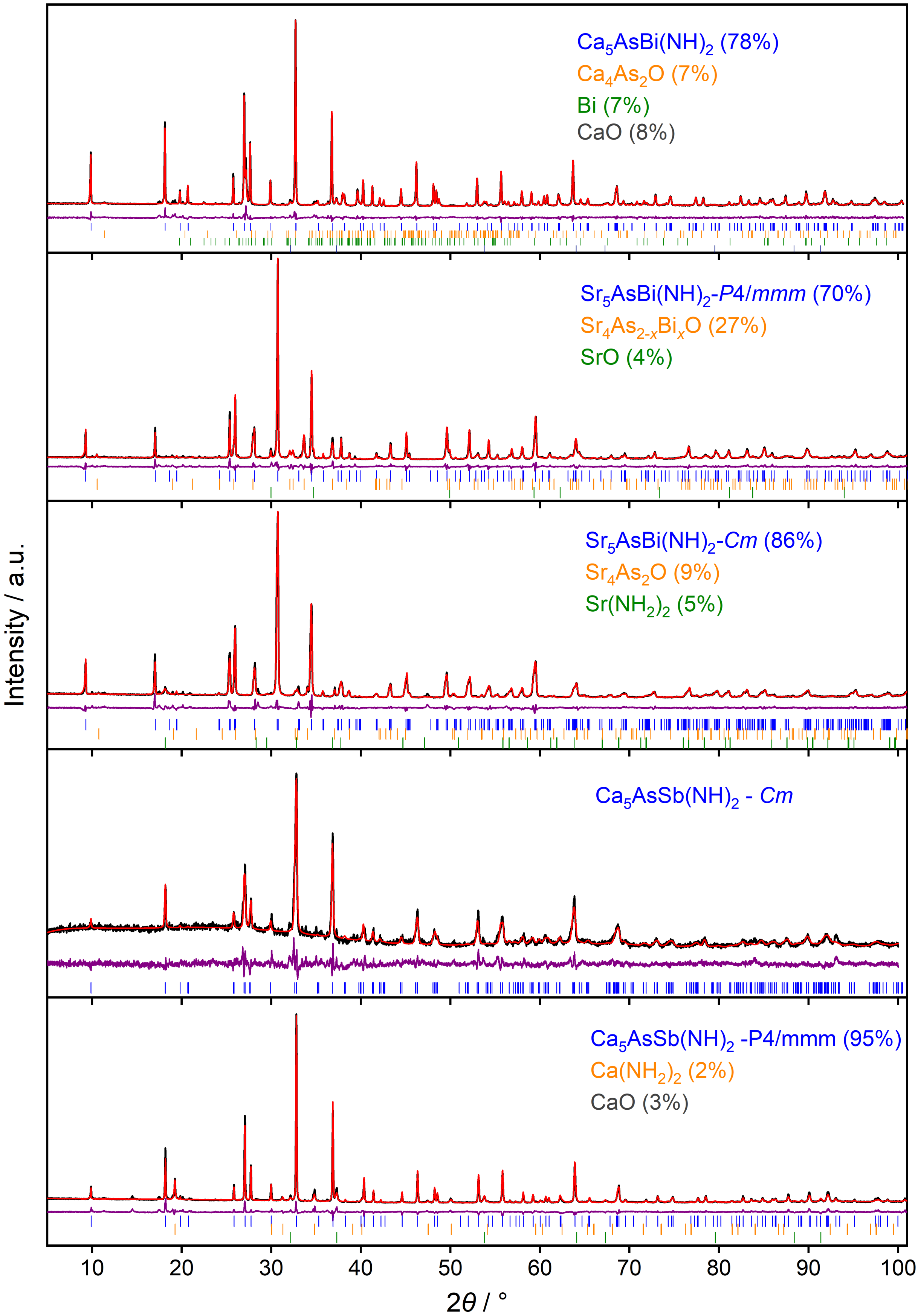

Figure S1: Measured and calculated diffractograms and difference profile of the Rietveld refinements of $Ca_5AsSb(NH)_2$ (*P4/mmm* and *Cm*), $Sr_5AsBi(NH)_2$ (*P4/mmm* and *Cm*) and $Ca_5AsBi(NH)_2$ (*Cm*) and byproducts.

Table S9: Selected distances in $Ca_5AsSb(NH)_2$ (*P4/mmm)*, $Sr_5As_{0.88}Bi_{1.12}(NH)_2$ (*P4/mmm*) and $Ca_5AsBi(NH)_2$ (*P4/mmm*) from scXRD data in Å.

formula	$Ca_5AsSb(NH)_2$	$Ca_5AsBi(NH)_2$	$Sr_5As_{0.88}Bi_{1.12}(NH)_2$	
N(1)-*AE*(1)	2.301(5)	2.299(5)	2.441(5)	1x
N(1)-*AE*(2)	2.4461(6)	2.4518(7)	2.6123(6)	4x
As(1)-*AE*(2)	3.1010(5)	3.0937(7)	3.2866(2)	8x
Pn(1)-*AE*(1)	3.4425(2)	3.4476(4)	3.6703(2)	4x
Pn(1)-*AE*(2)	3.5190(5)	3.5359(8)	3.7736(3)	8x

Table S10: Selected distances in $Ca_5AsSb(NH)_2$ (*Cm*) and $Sr_5AsBi(NH)_2$ (*Cm*) from pXRD data in Å.

formula	$Ca_5AsSb(NH)_2$		$Sr_5AsBi(NH)_2$	
N(1)-*AE*(2)	1x 2.35(8)	-	1x 2.462(4)	-
N(1)-*AE*(3)	2x 2.33(4)	2x 2.58(5)	2x 2.50(3)	2x 2.71(3)
N(2)-*AE*(1)	2x 2.27(4)	2x2.70(4)	2x 2.39(3)	2x2.85(3)
N(2)-*AE*(2)	1x 2.326(9)	-	1x 2.496(6)	-
Pn(1)-*AE*(2)	1x 3.21(8)	2x 3.457(5)	1x 3.58(7)	2x 3.672(2)
Pn(1)-*AE*(2)	1x 3.70(8)	-	1x 3.78(7)	-
Pn(1)-*AE*(1)	2x 3.43(5)	2x 3.69(5)	2x 3.612(3)	2x 3.73(4)
Pn(1)-*AE*(3)	2x 3.34(5)	2x 3.59(5)	2x 3.83(4)	2x 3.88(3)
As(2)-*AE*(1)	2x 2.89(5)	2x 3.24(5)	2x 3.27(4)	2x 3.42(4)
As(2)-*AE*(3)	2x 3.33(5)	2x3.09(5)	2x 3.23(4)	2x 3.27(4)

Table S11: Atomic coordinates, occupancy, Wyckoff symbol and equivalent displacement parameters ($Å^2$) of $Ca_5AsSb(NH)_2$ in *Cm* from powder X-ray diffraction data.

Atom	Wyckoff	*x*	*y*	*z*	U_{eq}	s.o.f
Sb01	2*a*	0.130(8)	½	0.410(3)	0.0112	0.864
As02	2a	0.129(8)	½	0.904(3)	0.0064	1
Ca03	4b	0.903(7)	0.739(4)	0.136(3)	0.0112	1
Ca04	2a	0.665(8)	½	0.39860	0.0112	1
Ca05	4b	0.416(8)	0.244(3)	0.698(3)	0.0112	1
N006	2a	0.61600	½	0.139	0.0228	1
N007	2a	0.61500	½	0.655	0.0228	1
H2	2a	0.64140	½	0.03320	0.0089	1
H3	2a	0.61010	½	0.76610	0.0089	1

Table S12: Atomic coordinates, Occupancy, Wyckoff symbol and equivalent displacement parameters (Å^2) of $Sr_5AsBi(NH)_2$ in *Cm* from powder X-ray diffraction data.

Atom	Wyckoff	*x*	*y*	*z*	U_{eq}	s.o.f
Bi01	2a	0.824(7)	½	0.6363(10)	0.0128	1
As02	2a	0.323(7)	0	0.131(2)	0.0106	1
Sr01	4b	0.092(6)	0.2512(13)	0.9094(13)	0.0154	1
Sr02	2a	0.338(7)	½	0.62099	0.0165	1
Sr03	4b	0.081(6)	0.2523(13)	0.3366(13)	0.0154	1
N001	2a	0.3132	½	0.36254	0.0108	1
N002	2a	0.2988	½	0.88183	0.0108	1

Table S13: Selected distances in Ca_3AsN, Ca_3SbN, Ca_3BiN, Sr_3AsN, Sr_3SbN and Sr_3BiN in Å.

formula	Ca_3AsN[23]	Ca_3SbN[24]	Ca_3BiN[24]	Sr_3AsN[23]	Sr_3SbN[24]	Sr_3BiN[24]
Crystal system	orthorhombic	cubic	cubic	orthorhombic	cubic	cubic
Space group	*Pnma*	$Pm\bar{3}m$	$Pm\bar{3}m$	*Pnma*	$Pm\bar{3}m$	$Pm\bar{3}m$
N-*AE*	2 x 2.391	6 x 2.427	6 x 2.444	2 x 2.391	6 x 2.586	6 x 2.603
N-*AE*	2 x 2.391	-	-	2 x 2.391	-	-
N-*AE*	2 x 2.391	-	-	2 x 2.391	-	-
Pn-AE	2 x 3.0682	12 x 3.457	12 x 3.457	2 x 3.1580	12 x 3.658	12 x 3.682
Pn-AE	1x 3.1601	-	-	1 x 3.1817	-	-
Pn-AE	1 x 3.2439	-	-	1 x 3.3186	-	-
Pn-AE	2 x 3,2742	-	-	2 x 3.4874	-	-
Pn-AE	2 x 3.4109	-	-	2 x 3.5466	-	-
Pn-AE	1 x 3.4849	-	-	1 x 3.9324	-	-
Pn-AE	1 x 3.5630	-	-	1 x 3.9919	-	-
Pn-AE	2 x 3.7286	-	-	2 x 4.2424	-	-

1.4 ^{1}H MAS NMR of *AE*$_5$As*Pn*(NH)$_2$

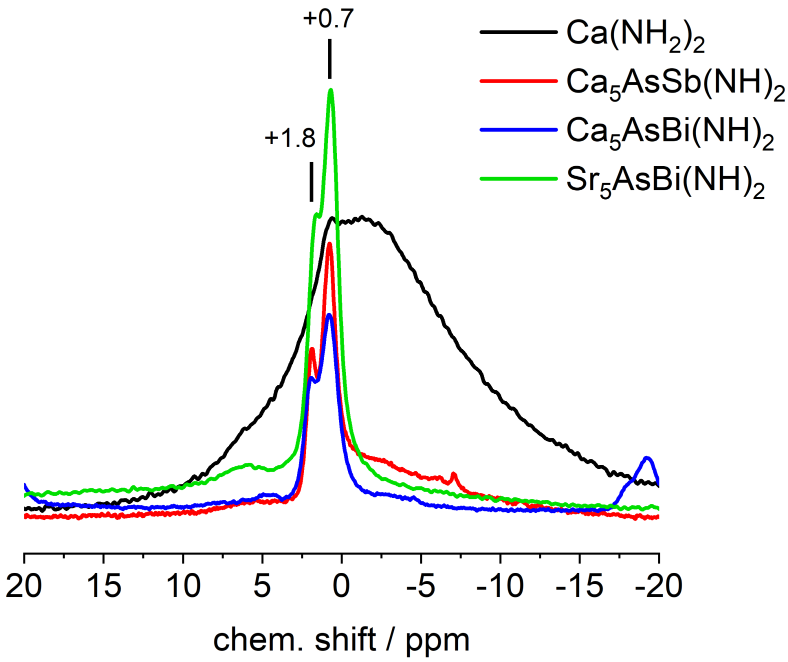

Figure S2: ^{1}H-MAS-NMR of $Ca(NH_2)_2$, $Ca_5AsSb(NH)_2$, $Ca_5AsSb(NH)_2$ and $Sr_5AsBi(NH)_2$.

1.5 IR-Spectra of $Sr_5AsBi(NH)_2$ (*Cm* vs. *P4/mmm*)

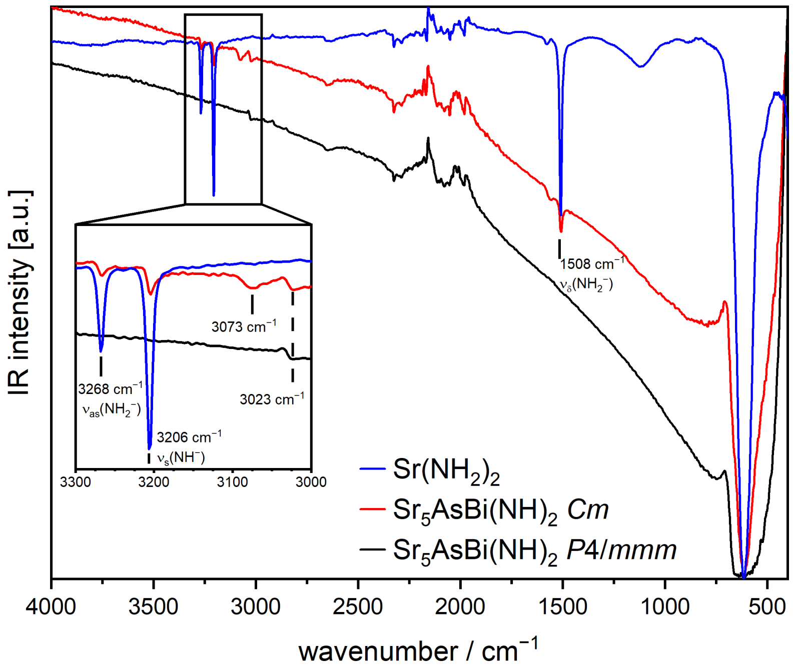

Figure S3: IR spectra of $Sr_5AsBi(NH)_2$ (*Cm* and *P4/mmm*) and $Sr(NH_2)_2$. Inset shows the NH vibration of $Sr_5AsBi(NH)_2$ (*Cm* and *P4/mmm*) and $Sr(NH_2)_2$ in the region between 3000-3150 cm^{-1}.

1.6 EDX-measurements

Table S14: Averaged EDX measurements.

formula	Ca (%)	Sr (%)	As (%)	Sb (%)	Bi (%)	Molar ratio *AE*: As: *Pn*
$AE_5AsPn(NH)_2$ (calc)	71.4		14.3	14.3		5: 1: 1
$Ca_5AsSb(NH)_2$	73(1)	-	13(1)	14(1)	-	5.57: 1: 1.06
$Ca_5AsBi(NH)_2$	71(1)	-	14(1)	-	15(1)	5.10: 1: 1.09
$Sr_5AsBi(NH)_2$	-	70.4(9)	14.3(5)	-	15.3(4)	4.93: 1: 1.07

2 Supplementary calculations

2.1 Convergence test of mobilities

Table S15: Convergence test of mobilities of $Ca_5AsSb(NH)_2$ in *Cm* under the condition of 300 K and electron carrier density of 1.0×10^{16} cm^{-3}. FIM represents the Fourier interpolated mesh for calculating transport properties.

	DFT: 12×12×7		DFT: 18×18×10	DFT: 20×20×11
	FIM: 65×65×35	FIM: 71×71×39	FIM: 97×97×53	FIM: 107×107×59
σ_{xx} (S m^{-1})	17.60	17.45	21.01	20.76
σ_{yy} (S m^{-1})	17.84	17.67	21.00	20.83
σ_{zz} (S m^{-1})	17.04	16.87	20.49	20.32

2.2 Electronic structure of $Ca_5AsSb(NH)_2$ and (b) $Sr_5AsBi(NH)_2$ in *Cm.*

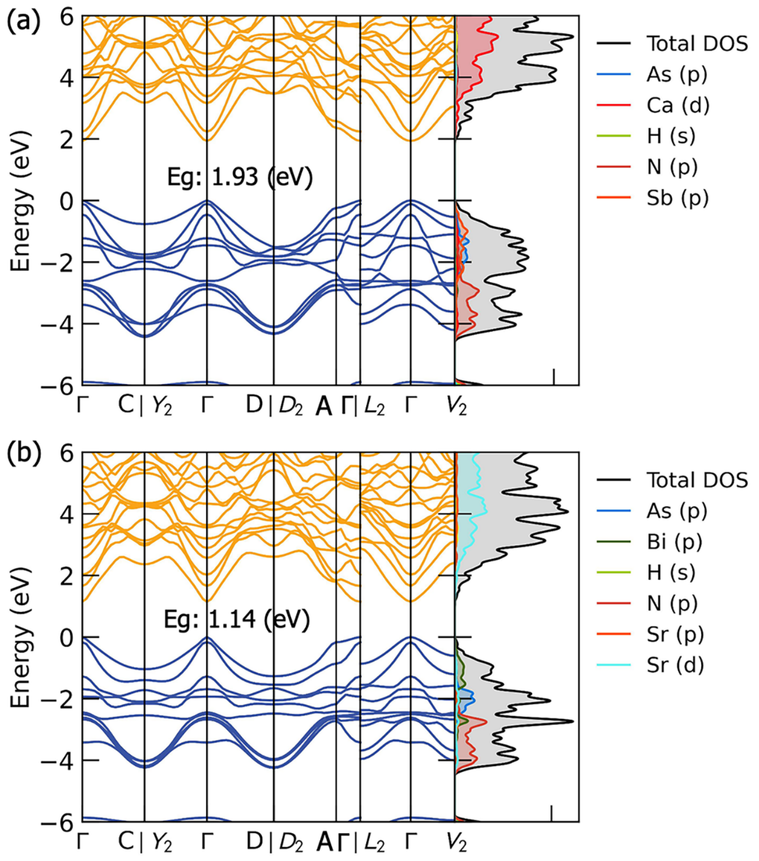

Figure S4: Band structure and density of states of (a) $Ca_5AsSb(NH)_2$ and (b) $Sr_5AsBi(NH)_2$ in *Cm* calculated at HSE+SOC level. The high symmetry k-points in the BZ zone areΓ:(0,0,0), C:(0.499,0.499,0.0), Y2:(-0.5,0.5,0), D:(-0.499,0.5,0.5), D2:(0.499,0.499,0.5), A:(0,0,0.5), L2:(0,0.5,0.5), L2:(0,0.5,0.5), V2:(0,0.5,0).

2.3 Calculated optical spectra of $Sr_5AsBi(NH)_2$

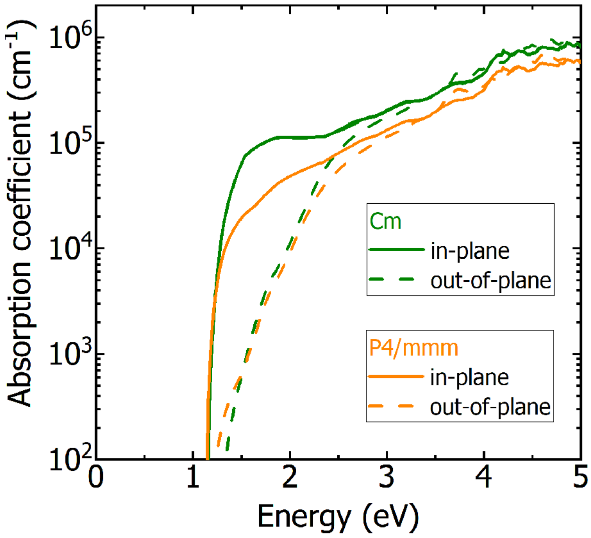

Figure S5: The calculated absorption spectrum of $Sr_5AsBi(NH)_2$ in *P4/mmm* and *Cm* symmetry.

2.4 IR spectrum of *AE*$_5$As*Pn*$(NH)_2$ calculated and experimental

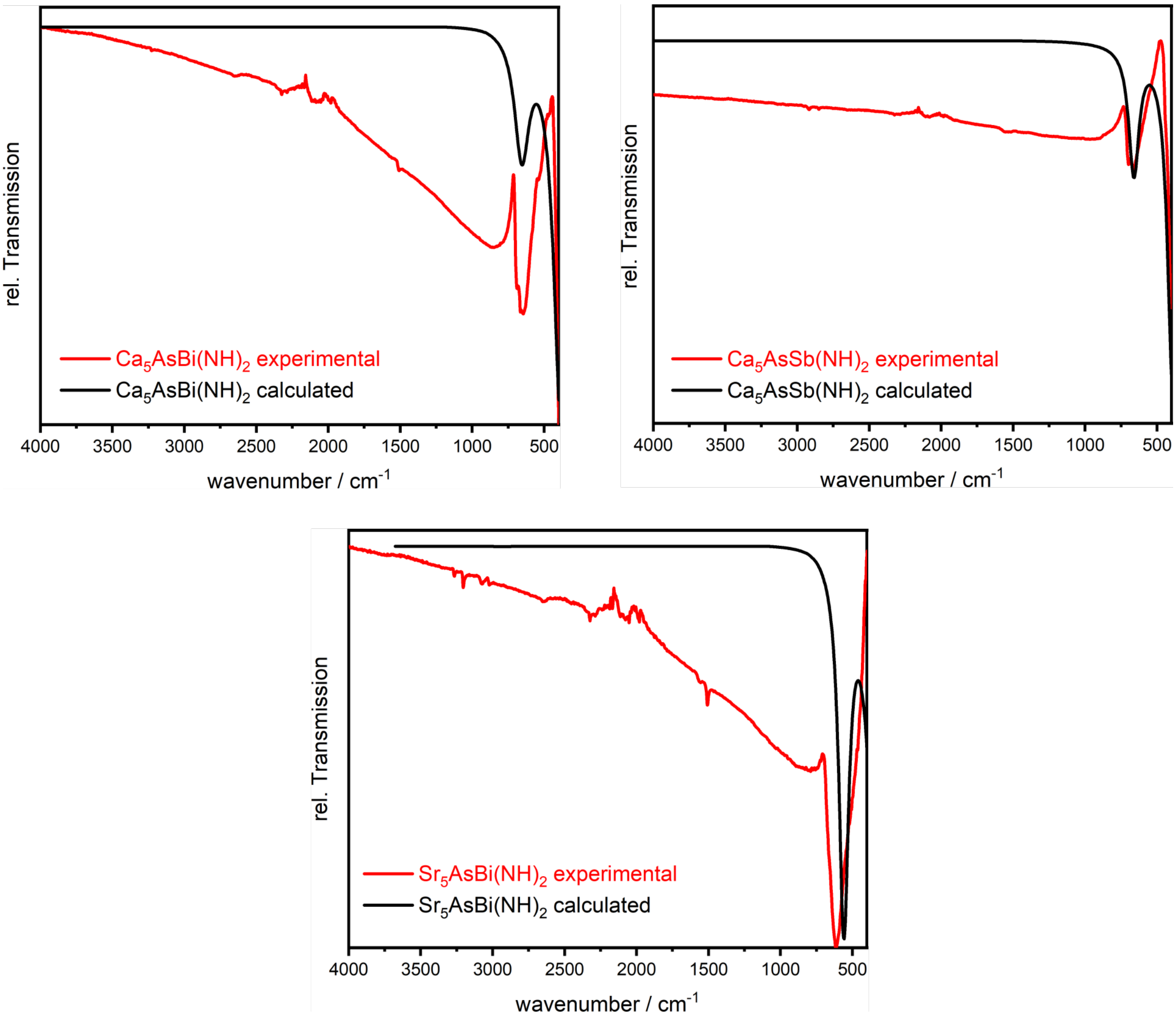

Figure S6: IR spectra of $Ca_5AsSb(NH)_2$, $Ca_5AsBi(NH)_2$ and $Sr_5AsBi(NH)_2$ in *Cm* symmetry.

[1] J. Häusler, W. Schnick, *Chem. Eur. J.* **2018**, *24*, 11864–11879.
[2] *APEX3*, v2016.5-0, Bruker AXS Inc, Madison, USA, **2016**.
[3] G. M. Sheldrick, *XPREP,* Version 2008/2; Bruker AXS Inc., Madison, USA, **2008**
[4] L. J. Farraugia, *J. Appl. Crystallogr.* **1999**, *32*, 837-–838.
[5] G. M. Sheldrick, *Acta Cryst. A* **2015**, *71*, 3–8.
[6] C. B. Hübschle, G. M. Sheldrick, B. Dittric, *J. Appl. Crystallogr.* **2011**, *44*, 1281-1284.
[7] A. Coelho, *TOPAS Academics, Version 6,* Coelho Software, Brisbane,Australia, **2016**.
[8] P. Kubelka, F. Munk, *Z. Technol. Phys.* **1931**, *12*, 593–601.
[9] G. Kresse, J. Furthmüller, *Phys. Rev. B* **1996**, *54*, 11169–11185.
[10] J. P. Perdew, K. Burke, M. Ernzerhof, *Phys. Rev. Lett.* **1996**, *77*, 3865–3868.
[11] J. Heyd, G. E. Scuseria, M. Ernzerhof, *J. Chem. Phys.* **2003**, *118*, 8207–8215.
[12] M.-H. Du, *J. Phys. Chem. Lett.* **2015**, *6*, 1461−1466.
[13] D. Han, M.-H. Du, C.-M. Dai, D. Sun, S. Chen, *J. Mater. Chem. A* **2017**, *5*, 6200-6210.
[14] A. M. Ganose, A. J. Jackson, D. O. Scanlon, *J. Open Source Softw.* **2018**, *3*, 717.
[15] U.-G. Jong, C.-J. Yu, J.-S. Ri, N.-H. Kim, G.-C. Ri, *Phys. Rev. B* **2016**, *94*, 125139.
[16] L. Yu, A. Zunger, *Phys. Rev. Lett.* **2012**, *108*, 068701-.
[17] A. M. Ganose, J. Park, A. Faghaninia, R. Woods-Robinson, K. A. Persson, A. Jain, *Nat. Commun.* **2021**, *12*, 2222.
[18] S. Baroni, S. d. Gironcoli, A. D. Corso, P. Giannozzi, *Rev. Mod. Phys.* **2001**, *73*, 515
[19] M. Rossi, M. Scheffler, V. Blum, *J. Phys. Chem. B* **2013**, *117*, 5574−5584.
[20] H. Shang, C. Carbogno, P. Rinke, M. Scheffler, *Comput. Phys. Commun.* **2017**, *215*, 26–46
[21] V. Blum, R. Gehrke, F. Hanke, P. Havu, V. Havu, X. Ren, K. Reuter, M. Scheffler, *Comput. Phys. Commun.* **2019**, *180*, 2175–2196.
[22] O. Lamiel-Garcia, K. C. Ko, J. Y. Lee, S. T. Bromley, *J. Chem. Theory Comput.* **2017**, *13*, 1785–1793.
[23] D. Stoiber, R. Niewa, *Z. Anorg. Allg. Chem.* **2019**, *645*, 329-334.
[24] R. Niewa, *Z. Anorg. Allg. Chem.* **2013**, *639*, 1699-1715.
